# Current Research on the Impact of Foreign Language Learning Among Healthy Seniors on Their Cognitive Functions From a Positive Psychology Perspective—A Systematic Review

**DOI:** 10.3389/fpsyg.2020.00765

**Published:** 2020-04-21

**Authors:** Blanka Klimova, Marcel Pikhart

**Affiliations:** Department of Applied Linguistics, Faculty of Informatics and Management, University of Hradec Kralove, Hradec Kralove, Czechia

**Keywords:** aging, older adults, foreign language learning, cognitive functions, positive psychology

## Abstract

The purpose of this review study is to explore the existing research focusing on the impact of foreign language learning among healthy seniors on their cognitive functions from the positive psychology perspective. The methods are based on a literature review of available sources found on the research topic in two acknowledged databases: Web of Science and Scopus. The search period was not limited by any time period since there are not many studies on this topic. Altogether seven original studies were detected. The findings of this review study thus reveal that foreign language learning (FLL) has a positive impact on the maintenance and/or enhancement of cognitive abilities irrespective of age. In addition, the FLL courses seem to offer new opportunities for healthy seniors in the area of socializing and integration into society, which consequently may positively affect their overall well-being. Furthermore, the research shows that it is partly through the stimulation of social well-being that the cognitive effects of FLL might be observed. Cognitive aspects of older age are to be further investigated, including the importance of learning a foreign language, as basically all research conducted proves at least some maintenance or even improvement of cognitive functions of older people when starting intensive language training.

## Introduction

The demographic situation of the so-called developed world can be described as a *rapidly aging population*. Consequently, in the coming decades the increase in the number of people in the age-group of older adults will be dramatic and unprecedented (Panitsides, 2014; Klimova, 2018). Additionally, incidences of dementia in developed countries have risen significantly and a similar global rise can be expected in the near future (Wong et al., 2019). In fact, at present, there are about 50 million people living with dementia (Klimova et al., 2019). The estimate for 2030 is 82 million demented people; in 2050, this number should reach 152 million (WHO, 2019). These facts have motivated researchers to look for ways of improving the quality of life of the older generation and to delay the onset of serious cognitive disorders such as dementia, of which Alzheimer's disease is the most common (Alzheimer's Society, 2019). Both pharmacological and non-pharmacological interventions have been studied with a variety of findings (van de Glind et al., 2013; Klimova et al., 2016). Unfortunately, the pharmacological strategies to treat the decline of cognitive functions have not yet produced satisfactory results (Klimova, 2018).

However, as research findings show (cf. Klimova and Kuca, 2015), there are several promising non-pharmacological strategies that appear to improve and/or maintain cognitive functions. These include physical activities, cognitive training, nutritious diet, as well as interventions for improvement of social interaction (cf. Ballesteros et al., 2015), including the use of modern information and communication technologies (cf. Ballesteros et al., 2014). One such cognitive training strategy seems to be FLL (Antoniou et al., 2013). Research has proved that foreign language training among older adults leads to improvement of cognitive functions (Wong et al., 2019). As Antoniou and Wright (2017) claim, cognitive performance can be boosted even at a later age by activities such as learning a foreign language and playing games. This happens because of the engagement of an extensive network in the human brain that overlaps with those brain parts that are most affected by the negative effects of aging (Antoniou and Wright, 2017). Multilingualism is a predictor of better cognitive abilities even in older generations and can delay the onset of serious mental illnesses, such as dementia, by a few years. This particular fact has been proven by research on bilingualism, which reveals that people who learn a second language in their adulthood may prevent cognitive decline in later life by ~4.5 years (cf. Bak et al., 2014; Bialystok et al., 2016). In addition, bilingualism represents a beneficial mental exercise for a large set of cognitive functions of the human brain (Bialystok et al., 2016).

Furthermore, it must be highlighted that aging dramatically influences fluid intelligence (i.e., the ability to reason and solve things) but does not affect crystallized intelligence (i.e., the ability to use knowledge and experience) (Klimova, 2018). Findings by Blumenfeld et al. (2017) indicate that older adults have better visual imagery and episodic memory than younger people.

In addition, learning a foreign language is considered to improve the quality of human life because, as proven by research, there is a definite correlation between life-long learning and the general well-being of a person (Pilar et al., 2014). Moreover, life-long learning, especially learning a foreign language, improves a person's social participation. Therefore, after a person's basic needs are covered, his/her general well-being can be improved through education-led personal improvement. Life-long learning, thus, touches upon a very important aspect, something that is inadequately addressed at the undergraduate level: education increases one's positive feelings about the self (Pilar et al., 2014; Narushima et al., 2018).

From a positive psychological perspective, FLL is not restricted to a young age—it is free of age-related constraints (cf. Seligman and Csikszentmihalyi, 2014). Current research into the impacts of bilingualism shows that it can boost a person's cognitive performance. However, bilingualism processes are bidirectional, i.e., language can impact cognitive functions and cognitive functions can impact language learning performance. Moreover, linguistic outcomes of third-age language learning are either not very important or they do not play a role at all (Pot et al., 2019).

Positive psychology holds immense promise for the improvement of positive emotions, well-being, and FLL (MacIntyre and Mercer, 2014). Learning is a process of acquiring new ideas, information, skills, and competencies with the eventual aim of attaining a state of knowledge. In this paper, the learning process focuses mostly on the journey, i.e., the process itself is sufficient when seen from a positive psychological perspective (Pot et al., 2019). The essential, and most positive, thing about the learning process is that the seniors are motivated to study a language for the sheer pleasure of learning (MacIntyre and Mercer, 2014), for the expressed purpose of learning another language and knowing about another culture, and for improving their social status as immigrants (Pot et al., 2018). The crucial point from a positive psychology perspective is that the outcomes of this learning process are significantly different from standard basic school or high school language education because in the older population the focus is on (1) the joy of lifelong learning, (2) their satisfaction with time well-spent in learning a new language, (3) their sense of belonging to a community of learners, and (4) the significant improvement of their cognitive functions, including memory, retention, and an enhanced sense of cognitive control (cf. MacIntyre and Devaele, 2014; Seligman and Csikszentmihalyi, 2014).

There are few studies on the effects of FLL on cognitive functions in old age (Antoniou et al., 2013). The most significant studies on this topic include those by Bak et al. (2016), Ware et al. (2017), Kliesch et al. (2017), and Wong et al. (2019)—the findings, however, are inconclusive. While Ware et al. (2017) claim that their research subjects found the program motivating and pleasant, they also admitted that scores in the Montreal Cognitive Assessment (MoCA)—a test designed to evaluate global cognitive functioning in older adults before and after instruction—did not differ significantly. On the contrary, Wong et al. (2019) proved that their computer-based language training software called Rosetta Stone had contributed to the improved cognitive abilities in healthy older Chinese. Bak et al. (2016) contend that even a short-term intensive language course is beneficial for participants' attentional functions.

The purpose of this review study is to explore the existing research focusing on the impact of FLL among healthy seniors on their cognitive functions from a positive psychology perspective.

## Methods

The method used was the review of literature on the topic available on two acknowledged databases: Web of Science and Scopus. The search period was not restricted because there are not many studies on this topic. The collocated keywords are as follows: *language learning* AND *healthy older people*; *language learning* AND *healthy seniors*; *language learning* AND *healthy older adults*; *language learning* AND *cognitively unimpaired older people*; *language learning* AND *cognitively unimpaired older adults; language learning* AND *cognitively unimpaired seniors;* and *language learning* AND *cognitively unimpaired elderly*. The keywords were combined and integrated into database and journal searches. The terms were searched using AND to combine the keywords listed and using OR to remove search duplication where possible. A backward search was also conducted, i.e., references of retrieved articles were assessed for relevant articles that authors' searches may have missed.

From the database/journal searches, 69 titles/abstracts were identified on the basis of the keywords: 62 in Web of Science and seven in Scopus. Another two articles were identified from other sources, mostly references of the already detected articles. After removing duplicates and titles/abstracts unrelated to the research topic, 55 studies—all in English— remained. Of these, only 22 articles were relevant to the research topic. These studies were thoroughly investigated and considered against certain inclusion and exclusion criteria.

The inclusion criteria were as follows:

- Only reviewed, full-text English studies in scientific journals were included.
- Only randomized controlled trials and experimental/cross-sectional studies were included.
- The primary outcome focused on the association between FLL in healthy seniors/older people/elderly and improvement and/or maintenance of their cognitive functions.
- The subjects were cognitively unimpaired older individuals of 55+ years.

The exclusion criteria were as follows:

- Descriptive studies (Panitsides, 2014); studies not focusing on the research topic, including the studies on bilingualism (Small et al., 1999; Friebe and Schmidt-Heartha, 2013; Clare et al., 2014; Zahodne et al., 2014; Narushima et al., 2018); studies having different age of the subjects (Schlegel et al., 2012; Bak et al., 2016; Ghazi-Saidi and Ansaldo, 2017); and review studies (Antoniou et al., 2013; Klimova, 2018) were excluded.

**Figure 1:**
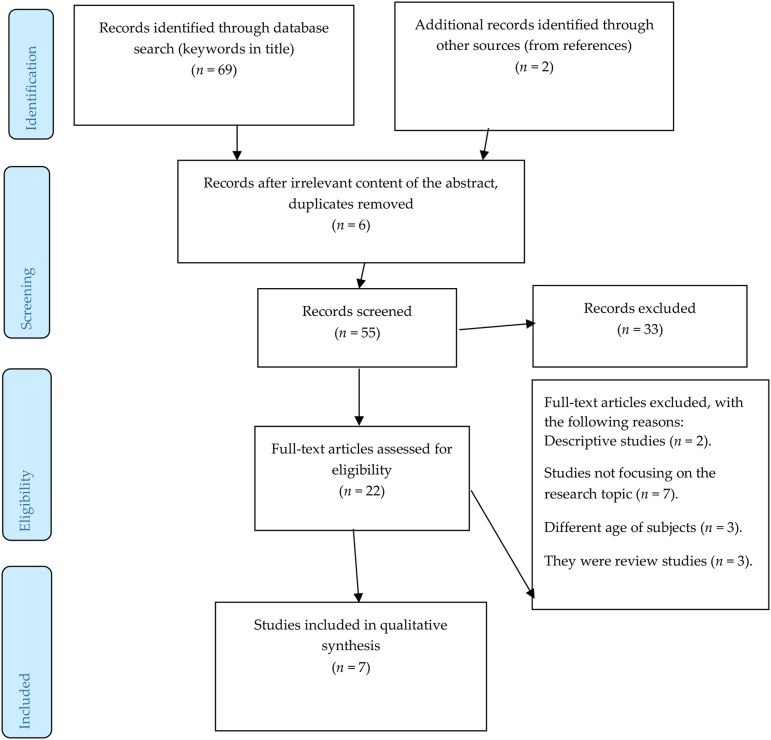
An overview of the selection procedure.

Based on these criteria, seven studies were included in the final analysis. Figure 1 illustrates the selection procedure.

## Results

Altogether seven original and relevant studies were detected. Two studies were randomized controlled trials (Bubbico et al., 2019; Wong et al., 2019) and five were experimental studies (Gruneverg and Pascoe, 1996; Kliesch et al., 2017; Ware et al., 2017; Pfenninger and Polz, 2018; Valis et al., 2019). Most of them originated in Europe: Austria, Czechia, France, Great Britain, Italy, and Switzerland (Gruneverg and Pascoe, 1996; Kliesch et al., 2017; Ware et al., 2017; Pfenninger and Polz, 2018; Bubbico et al., 2019; Valis et al., 2019) and one (Wong et al., 2019) was a joint research of China and Australia.

The main aims of these studies were to investigate whether FLL enhances cognitive skills among healthy older individuals; to detect functional changes in the brains of healthy seniors who have learnt a foreign language; to explore the effect of cognitive capacities on learning outcomes when learning a second language (L2); to investigate feasibility of a FLL course among healthy older people; and to test the keyword method for acquiring new L2 vocabulary.

The number of subjects in the above-mentioned studies ranged between 10 and 153 healthy older individuals. Except for one study (Pfenninger and Polz, 2018) where there were six German–Slovenian bilinguals, all subjects were monolingual native speakers. Apart from the study by Gruneverg and Pascoe (1996), whose subjects were studying Spanish, participants were studying English as a foreign language. In three studies (Gruneverg and Pascoe, 1996; Pfenninger and Polz, 2018; Wong et al., 2019) there were active control groups in addition to the experimental intervention groups. Two studies had passive control groups (Bubbico et al., 2019; Valis et al., 2019) and two studies did not have any control group. The intervention period ranged from 1 day to 6 months. All studies, except one (Gruneverg and Pascoe, 1996), were run onsite—most often in classrooms. As far as the teaching methods are concerned, four studies implemented different face-to-face approaches to FLL. Two studies (Ware et al., 2017; Wong et al., 2019) were computer-based and one study (Gruneverg and Pascoe, 1996) used a special self-study keyword method for acquiring new Spanish vocabulary. The key outcome measures usually included a battery of neuropsychological tests, foreign language assessment tests, and statistical analysis.

The findings of these studies revealed that FLL has a positive impact on the maintenance and/or enhancement of cognitive abilities (Gruneverg and Pascoe, 1996; Ware et al., 2017; Pfenninger and Polz, 2018; Bubbico et al., 2019; Valis et al., 2019; Wong et al., 2019), irrespective of age and bilingualism (Pfenninger and Polz, 2018), which is in contrast with another study (Kliesch et al., 2017). However, one has to be slightly critical of these results as some of the studies lacked control groups, some had passive groups, some employed qualitative analysis, and there were significant differences in the length of the intervention period. Despite these shortcomings, FLL courses appeared to be feasible and stimulating for healthy older people (Ware et al., 2017; Pfenninger and Polz, 2018; Valis et al., 2019) and positively affected their overall well-being, including their emotional well-being. Results showed that they felt optimistic and self-confident (Ware et al., 2017; Pfenninger and Polz, 2018; Valis et al., 2019). The participants also reported that they were proud of their families that supported them in their studies (Pfenninger and Polz, 2018; Valis et al., 2019). Furthermore, the research indicates that teaching methods such as group discussions, reading, playing games, watching YouTube videos, or singing in a foreign language can stimulate older adults learning a foreign language (Gruneverg and Pascoe, 1996; Kliesch et al., 2017; Pfenninger and Polz, 2018; Valis et al., 2019). Table 1 summarizes the key findings of the detected research studies, which are ordered alphabetically according to the surname of their first author.

**Table 1:** An overview of the research studies on the intervention of foreign language learning among healthy older individuals.

**Study**	**Objective**	**Characteristics of subjects (number, groups)**	**Length of the intervention (foreign language learned, learning settings, and teaching method)**	**Main outcome measures**	**Results**
Bubbico et al. (2019) RCT (Italy)	To analyse how second language learning program can contribute to functional changes in the brain of healthy older people.	Twenty-six old monolingual Italian native speakers, between 59 and 79 years of age; 14 people in the intervention group and 12 in the passive control group	Four months, 16 × 2 h sessions, the subjects were learning English, all were beginners. They were taught in class by a native speaker who implemented different face-to-face teaching methods to develop all language skills.	A battery of neuropsychological tests, MRI scanning, statistical analysis.	The results showed a significant improvement in global cognition together with an increased functional connectivity in the right inferior frontal gyrus (rIFG), right superior frontal gyrus (rSFG), and left superior parietal lobule (lSPL) among the subjects of the intervention group.
Gruneverg and Pascoe (1996) Experimental study (GB)	To explore the keyword method for both productive and receptive learning of foreign language vocabulary among elderly females.	Forty females speaking only English, divided into experimental and active control groups, age between 60 and 82 years.	Intervention period: one day; all subjects should have recalled individual basic Spanish words through the keyword method. There was no time limit for their recall. This was performed at home by self-studying and immediately after recalling 20 words, they were tested.	Test (productive recall).	The keyword method significantly enhances recall of the English word given its Spanish equivalent (receptive learning) and significantly enhances the learning of Spanish equivalents of English words (productive learning) using a “soft” criterion of correctness, compared to a control group given no instruction on how to learn.
Kliesch et al. (2017) Experimental study (Switzerland, Canada, Austria)	To investigate how general cognitive capacities affect the learning outcome of L2 training in older people.	Ten old monolingual German native speakers, between 65 and 74 years of age. No control group.	Sixty English lessons for beginners in 3 weeks. The subjects were taught in class by different face-to-face teaching methods to develop all language skills.	Language assessment test, cognitive tests, statistical analysis.	Cognitive fitness is an important factor in the variance of foreign language development as a function of L2 training in a school context. Being a bilingual helps in learning L2.
Pfenninger and Polz (2018) Experimental study (Austria)	To investigate cognitive benefits and feasibility of foreign language course among healthy older individuals.	Twelve German-speaking subjects, half of them German–Slovenian bilinguals in the experimental groups and six monolingual German speakers in an active control group, age: 63–90 years.	Four weeks, 2 h three times a week. The subjects were beginners, studying English in classroom setting. Both groups were taught by the same teacher who implemented different face-to-face teaching methods to develop all language skills.	Language assessment tests, cognitive tests, qualitative analysis, statistical analysis.	The learning of an additional language can contribute to healthy and active aging, as it has a positive effect on executive function (linguistic) self-confidence, autonomy, communication skills, and overall well-being, irrespective of age, and prior language knowledge (bilingualism).
Valis et al. (2019) Experimental study (Czechia)	To investigate the extent to which FLL may enhance cognitive functions among healthy older individuals.	Forty-two cognitively unimpaired monolingual Czech native speakers; 20 subjects in the experimental group and 22 in the passive control group; average age: 70.9 years.	Learning English for 12 weeks (three 45-min lessons per week). The participants in the experimental group were divided into a beginner group (9 people) and lower-intermediate group (11 people). They were exposed to different teaching methods in the classroom settings.	Cognitive assessments using standardized tests, intervention through English language teaching and learning, qualitative analysis, and statistical analysis.	The results of the research show that there has been a slight enhancement of cognitive skills in the experimental group. Nevertheless, on the whole, the scores of the experimental and control groups did not considerably differ.
Ware et al. (2017) Experimental study (France)	To examine the effect of teaching English using a computer program on cognitive functions, as well as its feasibility for healthy older people.	Fourteen monolingual French native speakers, average age 75. No control group. The participants had varying levels of English.	Four-month pilot study (16, 2-h sessions). It was an onsite technology-based program for learning English as a foreign language. It was run by an English speaking psychologist with experience in teaching English.	MoCA test, University of California Loneliness Assessment (UCLA), questionnaires, interviews.	The program is feasible, enjoyable, and stimulating, however, pre- and post-intervention scores of the MoCA did not significantly differ.
Wong et al. (2019) RCT (China, Australia)	To explore cognitive enhancing effect of FLL in older adults with no clear signs of cognitive decline.	One hundred fifty-three cognitively unimpaired monolingual Chinese native speakers, between 60 and 85 years of age. They were divided into three groups: two active groups and one passive, (FLL−53, games−51 and listening to music—passive control group−49).	Six months (5 h per week). The subjects participated in individual computer-based cognitively stimulating activities. In addition, each group had regular social activities held twice a months in their community centers.	A battery of cognitive tests, statistical analysis.	Results of FLL and games, but not music appreciation, improved overall cognitive abilities that were maintained.

## Discussion

As seen in Table 1, FLL has a positive effect on the maintenance and/or improvement of cognitive functions in healthy seniors irrespective of their age and bilingualism, although bilingual participants, compared to monolinguals, performed better at task-switching in a color–shape task (i.e., categorization of images by their color and shape). Furthermore, fMRI scans taken during this activity revealed a decreased activity in the bilinguals' left lateral frontal cortex and cingulate cortex—an indication of efficient executive functioning (Antoniou and Wright, 2017).

Wong et al. (2019) conducted the first randomized controlled study on the potential influence of foreign language acquisition in old age on a person's cognitive functions and the study offers crucial empirical findings. After a 6-month period of foreign language training, the authors could measure improved cognitive skills in the test group, and similar improvements in the control group, which focused on participation in games. Particularly, foreign language training resulted in improved working memory; the games, however, improved attention. The preliminary findings—even if the researched sample was relatively small—proved to be significant for further research to show whether FLL has an advantage over other activities connected to the improvement of cognitive skills in elderly people. The results highlight that intensive FLL, even at a later age, can yield statistically relevant results regarding working memory and cognitive function improvement. These findings are in agreement with other studies such as Borella et al. (2013) who claim—on the basis of their verbal working memory training program in old-old individuals aged 75+ years—that there is still room for plasticity in the basic mechanisms of cognition in advance old age. The same is true for the study by Buschkuehl et al. (2008). In addition, Buitenweg et al. (2012) emphasize that for successful brain training among healthy seniors it is important to focus on memory strategy training, important to tailor the training to the needs of each individual, and include flexibility and novelty in the training.

Valis et al. (2019) do not confirm a significant improvement in the cognitive functions in elderly people because of FLL. Instead they confirm the more realistic *maintenance of cognitive skills* rather than their improvement. Intuitively, as well as supported by various studies, we can accept that FLL will have some positive impact on cognitive skills. However, the most important question is that to what extent it will influence the cognitive skills in the older population. The research results do not confirm the hypothesis that the learning of a foreign language will naturally enhance cognitive skills. Nevertheless, they yield significant findings suggesting a possible impact of FLL on the maintenance of cognitive skills, i.e., there was, at least, no observable decline of cognitive functions in the test group. Regular cognitive training, such as FLL, thus, will not function as a way to improve cognitive skills but rather as an efficient tool for their maintenance. Therefore, FLL can sufficiently contribute to a non-pharmacological strategy in preventing the onset of cognitive decline.

Furthermore, short-term language training in healthy seniors can lead to a significant improvement in global cognition with increased functional connectivity in the right inferior frontal gyrus, the right superior frontal gyrus, and the left superior parietal lobule. These findings are part of the current neuroscience breakthroughs that reshape neural networks through foreign language training. Bubbico et al's (2019) recent study indicates that a 4-month language course of English in Italian seniors improves global cognitive functions and reorganizes functional cognitive connectivity. It was only the intervention group that yielded significant improvement results in both functional and behavioral measures after the intervention of the language class. In addition, FLL studies conducted among adult learners show that certain teaching methods, such as repetition, imitation, and drilling, can have a positive impact on higher network configuration (Ghazi-Saidi and Ansaldo, 2017).

The results of this review study also reveal that FLL in older adults contributes to establishing strong social ties between the participants, promoting social interaction and integration. Naturally, most language classes are held in classrooms, facilitating regular physical meetings of participants. This helps to develop social connections among participants and instills in them a sense of participation. The older generation often suffers from social isolation, which can possibly be an important factor of lower level of well-being and a source of depression (Popa-Wagner et al., 2014; Sandu et al., 2015). Therefore, language classes may not just improve or maintain cognitive functions but also create an environment of social meetings and networking (cf. Diaz-Orueta et al., 2012). As Narushima et al. (2018) point out, continuous participation in life-long learning courses, such as foreign language courses, can develop social cohesion and improve the sense of community participation, which may lead to improved social performance and enhanced well-being. Pot et al. (2018) expand that it is partly through the stimulation of social well-being that the cognitive effects of FLL might be observed.

In light of expected global demographic changes, the established cognitive and mental benefits of FLL in old age, and the role of FLL in the improvement of general human well-being, universities should offer relevant courses for the third-age learners. The breakthrough research by Singleton and Pfenninger (2018) moves our attention to the importance of language learning in old age. Their study highlights an abundance of research into second-language education in young people and a dearth research regarding the importance of such education in old people. They also confirm the hypothesis that FLL is a promising way to healthy and active aging because of its many advantages. Moreover, according to them the traditionally accepted approach that early exposure to foreign language necessarily brings better results than late exposure has not been proven. Early beginners did not necessarily outperform late beginners, therefore, this widely and intuitively accepted premise is not necessarily true and needs further investigation. Some research into specific learning needs of elderly students has been conducted and the findings are important. Take for example the research of Gruneverg and Pascoe (1996) into the efficiency of the keyword method for foreign language vocabulary learning in the elderly.

Yi-Yin (2011) researched the motivation paradigms in the older generation and concluded that they are rather different from the younger generation. The most important drives are desire for knowledge, desire for stimulation, desire for self-fulfillment, and desire for generativity. These findings are significant for the creation of curricula of the universities of the third-age creators.

Moreover, there is an emerging trend within learning psychology and related disciplines—the use of mobile technologies in the learning process. Again, there is a plethora of literature on the use of technology in basic, high school, and university education but little on the use of technological tools by older learners. Some introductory experimental findings are, however, described by Ware et al. (2017) and Wong et al. (2019). Additionally, the authors suggest future directions for the use of technology in FLL as a therapeutic and cognitive intervention.

Further pedagogical implementations of the findings will be important. Moreover, pedagogy and psychology of learning ought to try to find new pragmatic approaches and methodologies in the light of both expected global demographic changes and the possible positive outcomes of FLL in old age. There is immense scope for FLL in old age and we need to create courses that will incorporate earlier research findings with anticipated future developments in the field Kliesch et al. (2017).

It is important to highlight that even authoritative textbooks on psycholinguistics such as Traxler's *Introduction to Psycholinguistics. Understanding Language Science* (Traxler, 2012) totally ignore the area of language acquisition in old age, focusing entirely on language development in infancy and early childhood. A similar thing can be observed in *An Introduction to Psycholinguistics* by Steinberg and Sciarini (2006) where some references to old age and FLL can be found, but it is rather insufficient and unrepresentative of research in the area. Similarly, other authoritative textbooks on cognitive linguistics do not address the aspect of old age and/or do not take it into consideration—or do so only fleetingly (Ungerer and Schmid, 2006). It is for these reasons that this review attempts to bring this matter to the attention of scholars and researchers, so that research effort into the issue could be improved. The authors of this study believe that the findings of this research will expedite the implementation of the results into reality as the way to the sustainability of our society and also our global competitiveness.

The above-mentioned research has several limitations. The findings are usually not very systematic, the author applies different methodologies, and the results are difficult to replicate and difficult to verify by further research (cf. Melby-Lervag and Hulme, 2013). However, the data provided is relevant and presents a significant view of the multifaceted aspects of healthy aging. Naturally, cognitive aspects of old age need to be further investigated—including the importance of learning a foreign language—because almost the entire body of research conducted on the topic shows some degree of maintenance—in certain cases even improvement—of the cognitive functions of older people when they start intensive foreign language training.

## Conclusion

The findings of this review study thus reveal that FLL has a positive impact on the maintenance and/or enhancement of cognitive abilities irrespective of age. In addition, FLL courses seem to offer opportunities to healthy seniors for socialization and integration into society, which may positively affect their overall well-being. Furthermore, research shows that it is partly through the stimulation of social well-being that the cognitive effects of FLL might be observed.

The topic of FLL in old age has attracted widespread attention of scholars in the past few years. The topic holds great promise for further development especially in the identification, analysis, and development of learning strategies to maintain—or even improve—cognitive and psychological aspects of older people's life. Foreign language education for older generation might offer crucial non-pharmacological strategies for healthy aging—a topic of utmost importance due to the negative demographic trends both in Europe and in the so-called developed world. However, further research is needed.

From a positive psychology perspective, future research should focus on what kind of positive instruction should be given to older adults so that we can understand them in a better way. It is also important to develop methodologies to improve old people's sense of accomplishment in the learning process. Last but not least: investigating how to improve the pleasure of learning in the older generation of learners because it is one of the most important aspects of their language education.

## Data Availability Statement

All datasets generated for this study are included in the manuscript.

## Author Contributions

BK and MP drafted, analyzed, wrote, and read the entire manuscript.

## Conflict of Interest

The authors declare that the research was conducted in the absence of any commercial or financial relationships that could be construed as a potential conflict of interest.
